# Healthcare Experiences and Needs of Patients with Pregnancy of Unknown Location: A Qualitative Study

**DOI:** 10.21203/rs.3.rs-6735016/v1

**Published:** 2025-06-10

**Authors:** Kavita Vinekar, Zahava Shimshi, Amanda Doty, Samantha Kolowrat, Rebecca Mercier, Agatha Berger, Kristin Rising

**Affiliations:** Thomas Jefferson University; Thomas Jefferson University; Thomas Jefferson University; Thomas Jefferson University; Thomas Jefferson University; Thomas Jefferson University; Thomas Jefferson University

**Keywords:** pregnancy of unknown location, early pregnancy, first trimester bleeding, pregnancy loss, pregnancy complications

## Abstract

**Background:**

Patients presenting with bleeding or pelvic pain in early pregnancy are commonly found to have pregnancies of unknown location (PUL), with no sonographic findings of intrauterine nor ectopic pregnancy. We aimed to explore healthcare experiences and needs of patients presenting with PUL.

**Methods:**

This was a qualitative study using semi-structured interviews to explore healthcare experiences among patients with PUL. Patients were eligible if they had a PUL followed in an electronic health record tracking list; had an initial human chorionic gonadotropin (hCG) level of > 5 mIU/mL; spoke English; and were 18 years or older. Quasi-inductive directed content analysis was performed using NVivo 13.

**Results:**

We conducted 20 qualitative interviews from November 2023 to March 2024. Sixty percent (n = 12) of participants identified as non-Hispanic Black, all identified as cis-gendered women, and the mean age was 30.6 years. All participants were insured and most (65%) had public insurance. Analysis resulted in findings around three general domains of PUL healthcare experiences: *provider communication, care environment and timely access to necessary services*. Participants generally appreciated a resident-staffed phone line for discussing questions and results. They noted successes and gaps in provider communication of uncertain diagnoses, mental health resources, and optimization of care environment, perhaps in a dedicated pregnancy emergency care unit.

**Conclusions:**

In facing the diagnostic uncertainty of PUL, patients voiced needs for more direct communication, more timely care and support resources, and more targeted pregnancy-specific emergency care. Our findings reveal opportunities for improvement in provider training and healthcare organizational structuring to better meet patient needs.

## Background

Around 25% of pregnant individuals experience early pregnancy vaginal bleeding or pelvic pain (1), often leading them to seek care in the emergency department (ED) or an early pregnancy assessment clinic (EPAC), which is an outpatient setting designed to fulfill clinical needs in early pregnancy (2–4). As ruptured ectopic pregnancy is the leading cause of first trimester maternal death in the United States(5), clinical protocols in early pregnancy are focused on rapid detection and management of ectopic pregnancies to prevent associated morbidity and mortality (6). However, these efforts at early detection of ectopic pregnancy may result in equivocal ultrasound findings with no definite intrauterine or extrauterine pregnancy, either because it is too early to confirm pregnancy location with ultrasound or because of a completed pregnancy loss. These are called pregnancies of unknown location (PUL) (7). Given the elevated risk of ectopic pregnancy in patients with PUL, management may include serial β-hCG levels, uterine aspiration, systemic methotrexate, laparoscopy, or repeat ultrasound.

The negative impact of diagnostic uncertainty on patient experience has been documented across other patient conditions (8–12), but little attention has been given to the needs and experience of patients with PUL. One prior qualitative study by Wu et al focused on patients with desired pregnancy experiencing PUL and found that patient priorities centered around the health of the pregnancy, their own health, future fertility, and diagnostic uncertainty, among others (13). To date, however, the lived experiences and needs of pregnant patients navigating diagnostic uncertainty with PUL are not well understood.

To that end, the goal of this qualitative study was to explore the experiences and healthcare needs of patients with PUL, with a specific focus on understanding experiences with diagnostic uncertainty. Findings from this work can be used to optimize patient communication and care delivery in the setting of a PUL diagnosis.

## Methods

This qualitative interview study was conducted at an urban academic center in Philadelphia, Pennsylvania. We identified all individuals who were followed and discharged from an electronic health record (EHR) tracking list of patients with PUL and ectopic pregnancies (‘Beta Book’) across two hospitals within one healthcare system between January 1, 2022, and December 31, 2023. Patients were eligible if they had an initial hCG level of >5 mIU/mL, were proficient in spoken English, and were 18 years or older at the time of initial care presentation, with these criteria assessed by chart review prior to recruitment. Patients were excluded if they met criteria for diagnosis of ectopic pregnancy or intrauterine pregnancy (irrespective of viability) in the initial clinical encounter, or if they had an incidental early pregnancy diagnosis with no symptoms of bleeding or pain. Eligible participants were contacted by phone for a total of three attempts. We planned for a target sample of 20–30 participants based on previous literature in this field (13), with enrollment continued until thematic saturation was reached (14).

We developed a semi-structured interview guide with questions designed to explore each participant’s needs, desires, and perceptions of healthcare engagement in the context of PUL. We explored the following domains: general healthcare experience, clinical setting, diagnostic uncertainty, role of race/ethnicity, phone interactions, pregnancy desiredness, and lab/imaging visits (*Appendix A*). Guide development was informed by the team’s clinical experience, prior research, literature review and expert consultation. The interview guide was piloted internally amongst team members and was iteratively revised.Verbal consent was provided on the phone during enrollment. Two co-authors (ZS and SK), trained in qualitative research methods, conducted all interviews and administered a demographic questionnaire to participants via secure video conferencing. Interviews lasted approximately 20–30 minutes and were audio recorded. All participants received a $20 payment card upon study completion.

Audio recordings were transcribed verbatim by an independent third party. Transcripts were checked for accuracy, deidentified, and analyzed using a quasi-inductive coding approach in NVivo 13 by members of the research team. Thematic codes emerged through two methods: one based on prior knowledge (guided by existing literature and interview prompts) and another through meticulous line by line examination of select interview transcripts. All research team members read the first three transcripts and identified concepts to develop the initial code structure. Each code was explicitly defined to ensure coding accuracy and to improve intercoder reliability. This initial code structure was applied to subsequent interviews by two coders (ZS and KV) and refined to include new themes as they emerged. 45% of transcripts were double-coded. Intercoder reliability was calculated in NVivo 13 using a Kappa coefficient threshold of 0.7. A constant comparison approach was used, whereby completed interviews were coded before later interviews were conducted. Coding was supervised by a qualitative research project manager (AD).

We collected participant age, race/ethnicity, marital status, insurance (commercial vs. public), residential address, pregnancy history, PUL pregnancy outcome, and questions about PUL experience including presenting location, presenting symptom(s), number of office visits, ED visits, ultrasounds, and blood draws through chart review and patient report. We obtained zip code data on median annual household income and poverty rates from the 2022 American Community Survey (U.S. Census Bureau, 2022). Using Google maps web application, we calculated travel distance and time (via car and via public transport) from residential address to presenting location (EPAC or ED) (*Google Maps*, 2024). We calculated descriptive statistics to summarize demographic information about the study population.

### Ethics approval and consent to participate

This study was reviewed and determined to be exempt by the Thomas Jefferson University Institutional Review Board (study #2023–2300). Informed consent was obtained from each participant prior to study participation.

## Results

Of 217 total patients included in the EHR PUL list, 173 met eligibility criteria, 73 were contacted, and 20 were ultimately enrolled and interviewed from November 2023 to March 2024 (*Appendix B*). Of the 20 study participants, the mean age was 30.6 years, all identified as cis-gendered women, and 12 (60%) identified as non-Hispanic Black. All patients were insured and 65% had public insurance. While participants were not asked their household income, zip code data showed that, among all participants, the median zip code percentage of people living under the federal poverty line was 27.2%, compared to a city-wide rate of 21.7% living under the federal poverty line (Table 1).(15)

Sixteen participants (80%) had experienced a pregnancy prior to the PUL. Fourteen participants (70%) had EHR documentation of the PUL being desired, 1 was undesired, 1 was unsure, and 4 were not clearly documented. Pregnancy outcomes of the PULs included two live births, one ongoing viable pregnancy at time of interview, one ectopic pregnancy treated with methotrexate, seven early pregnancy losses, and nine spontaneous resolutions of the PUL without confirmed location.

Most participants (80%) initially presented to the ED; the remaining 20% presented to the EPAC. The most common primary reason for presenting was vaginal bleeding (70%), pain (15%) and desire to confirm pregnancy after a positive home pregnancy test (10%). Beyond the initial PUL encounter, all participants engaged with a dedicated patient-facing resident-run “Beta Book phone”, available 24 hours per day, 7 days per week to all patients with PUL. Participants were followed on the “Beta Book” for an average of 23.3 days.

Interview analysis resulted in three overall themes related to healthcare experiences of patients with PUL: *provider communication*, *timely* access to necessary services, and *care environment* (Table 2).

### Provider Communication

Participants often discussed provider communication around the PUL diagnosis. Many were confused about the medical details presented to them. Others acknowledged that, while the provider communication was sufficient, the inherent uncertainty of a PUL overshadowed the provider interaction. Themes of insufficient communication and the emotional toll of uncertainty emerged prominently. Prior pregnancy experiences, including losses, played a significant role in shaping participants’ expectations and reactions, highlighting the need for more personalized and empathetic care. Overall, providing education on PUL and the challenges of diagnostic uncertainty positively influenced patients’ perceptions of their interactions and emotional connection with their providers.

One theme that arose was that the communication of uncertainty around the PUL diagnosis led to a sense of false hope for a pregnancy that was likely nonviable. As one patient said, “…I’m just sitting there dumbfounded, confused, clueless, and then still hoping, despite my tears, that I’m still potentially pregnant when I wish someone would have been preparing me for the fact that more than likely I’m not.” One participant described confusion stemming from the dissonance between the seriousness of a potential ectopic pregnancy diagnosis and the time required for follow-up blood work, attributing this to a lack of communication regarding the purpose, timing, and anticipated outcomes of these tests: “It didn’t really make sense to me that if they thought [it was an ectopic pregnancy] that, like, it wasn’t like an immediate emergent thing that I had to get done. They just kept sending me to get bloodwork. Like, I was getting bloodwork, I think, every two days.” Participants often felt they lacked closure in their PUL experience and were forced to draw their own conclusions about the pregnancy outcome. One participant who had a spontaneous resolution to the PUL noted: “I had to come to the conclusion on my own that I had, you know, lost the baby. No one actually said, ‘you just had a miscarriage’.”

Participants also noted that the communication felt indirect or evasive. One reported, “…I feel like the doctors more so talked outside of the room, versus more to me.” Some felt that while providers offered resources, they did so without genuine concern: “[I wanted] the doctors to show that they care more and be more helpful and not just throw the resources at us, like here’s the paper.”

Although insufficient communication emerged as a theme, many participants expressed feeling heard and supported by their providers and reported receiving the education necessary to make informed medical decisions at the time: “I never knew about an ectopic pregnancy or, you know, things like that. Like I was all kind of new to it, but the doctors were supportive and, you know, definitely telling me my different options”. When providers incorporated education on PUL and diagnostic uncertainty into their discussions, patients perceived this as a positive aspect of care, improving their perception of the interactions with providers.

Several participants experienced prior pregnancy losses or ectopic pregnancies. They noted the importance of these previous pregnancy experiences in informing the communication around their PUL experience. In some cases, the PUL experience triggered traumatic memories of a prior pregnancy loss. Many expressed a desire for their healthcare provider to acknowledge recurrent pregnancy complications. Others noted that their previous pregnancies had been healthy, and they were emotionally unprepared for a pregnancy complication.

Participants reported a range of experiences around providers exploring their pregnancy intentions and desires. Several had concerns about their future fertility and felt reassured when this was acknowledged: “It’s not like they told me to stop trying [to conceive] because that would have broke my heart.” Some noted that providers made assumptions about their pregnancy desires, at times assuming that a pregnancy was desired (one participant was congratulated on her new pregnancy diagnosis) and at other times assuming the opposite: “It just felt like they were like moving towards termination like without me even saying that’s what I wanted.”

### Care Environment

Participants discussed preferences for initial care setting and what influenced their decision to present either to the ED or EPAC. The most common reasons for choosing where to seek care included prior personal or family experience with a specific healthcare network, Internet searches, and close vicinity. While participants often expressed a desire for specialized early pregnancy services, many were unaware of the EPAC services available and instead presented to the ED.

Several patients voiced a desire to see an outpatient OB/GYN earlier in the pregnancy while others and were not informed of EPAC services despite calling the OB/Gyn office for an appointment. Furthermore, many expressed distress about access to outpatient care, noting that the challenge of scheduling an OB/GYN appointment compounded their grief and uncertainty. One participant who ultimately presented to the ED for initial pregnancy care noted, “I was not able to really get an appointment until, like, the eight-week point, um, so you know, obviously I would have liked to have gotten in sooner or as soon as I – I realized I was pregnant, which I found out at, like, the 4.5-week, um, point. Like as soon as I missed my period, I knew I was expecting, so it would have been nice to have gotten in, you know, as soon as possible.”

A few participants expressed the need for the physical environment to be improved, particularly in the ED, citing a need for a more inviting and hygienic environment. One participant described the unsanitary and chaotic conditions of her ED visit, noting fecal matter and urine on the chairs and a sense of chaos in the waiting room. Some participants expressed the desire for OBGYN related emergencies to be addressed in another area of the hospital, outside of the ED.

### Timely Access to Necessary Services

Participants highlighted several critical issues concerning timely access to necessary services in PUL management, especially in the ED. Patient goals and concerns for their care included wanting to confirm pregnancy following a positive home pregnancy test, to have an assessment for possible ectopic pregnancy or miscarriage, and to address concern over having a successful pregnancy. The perceived lack of urgency, long wait times, and lack of updates from medical staff were emotionally distressing for some participants. Many commented on the need for more immediate and timely counseling and grief support, which significantly influenced the patient experience.

Participants expressed frustration with the perceived lack of urgency in emergency medical care, contrasted with the counseling they received about the risks of miscarriage or ectopic pregnancy. While in the ED, patients felt their concerns, such as the acuity of their situation, pain, and fear of future infertility, were not being addressed in a timely fashion: “And if something is that risky and that fatal to somebody, and that could be something that is like a cause of death to somebody, or like cause infertility, I feel like those should be like prioritized more.”

This sense of being overlooked was echoed by many participants who expressed concerns regarding long wait times and lack of updates in the hospital’s ED. One participant said it took them four to five hours to even be told they were pregnant, and then they had to leave the ED due to other time constraints. Several participants found the wait time to be emotionally distressing. One participant suggested more frequent communication about anticipated wait time. Others recommended opening an ED triage section specifically for early pregnancy evaluation or allowing PUL care to be managed in the OBGYN department.

Participants reported a general appreciation for having direct access to a resident-staffed secure line that they were able to text or call directly: “I think that was it was helpful again just having someone to kind of explain the process and follow up. And, um, it definitely, uh, felt, you know, um, that my care was being followed adequately. “ While most felt it was accessible and had positive interactions with the residents, other participants had neutral experiences with the Beta Book phone, and one noted feeling overwhelmed by the frequent contact and reminders and stopped responding to the calls and text messages.

Some participants mentioned the need for improved mental health support, noting a need for counseling or trained social workers to help address early pregnancy distress. Others noted that they weren’t seeking emotional support from their provider because they had that in a partner or family member and preferred to keep the healthcare interaction as brief as possible.

## Discussion

In this qualitative study exploring healthcare experiences and needs of patients with PUL, we identified three major themes: *provider communication, care environment*, and *timely access to necessary services*. Patients overall desired more open and comprehensive communication from providers and identified a need for more timely access to care in early pregnancy. This includes expedited access to early pregnancy ambulatory care for scheduling outpatient appointments (e.g., through the EPAC) and for emergency care, such as within a dedicated hospital-based pregnancy unit. In subsequent healthcare encounters, they reported an overall appreciation for direct communication with a resident-staffed phone line.

This study adds to a growing body of literature around patient experiences in early pregnancy. Our literature search revealed one prior qualitative study of PUL experiences by Wu et al (13). All participants in that study had desired pregnancies and approximately one-third were recruited from a subspecialty fertility clinic setting. Our study built upon these findings in a different population of individuals experiencing PUL and overlapped in several themes including health of pregnancy and self, future fertility, and mental health support. Prior qualitative studies of patient experiences with miscarriage have revealed themes including a lack of prior knowledge and experiences with miscarriages, insufficient information from ED providers about why the miscarriage was happening, what could have been done to prevent a pregnancy loss, and what it meant for future fertility, and a lack of emotional support (17,18). Many felt unprepared to face a miscarriage and were uncertain about what to expect going forward. Our study reflects similar findings, with an additional element of diagnostic uncertainty that further clouds communication and often left participants feeling as though they needed to draw their own conclusions about the final diagnosis.

Our finding of the patient-reported need for improved communication at the time of PUL diagnosis expands upon prior work in which emergency medicine providers reported the need for additional training for effective communication in the setting of first trimester bleeding and pain, and diagnostic uncertainty (19). Developing a patient-centered approach to communication in the context of PUL and subsequent training of emergency and gynecology providers in this approach could enhance patient experience by providing clearer information about pregnancy prognosis, diagnostic conclusions, and future fertility.

The 2022 U.S. Supreme Court decision in *Dobbs* vs *Jackson Women’s Health Organization* granted states the unrestricted power to ban abortion care, with far-reaching implications for early pregnancy care. The *Dobbs* decision was not mentioned in the interview guide, nor did it come up in interviews. Of note, this study was conducted in Pennsylvania, where abortion remains legal through 23 weeks 6 days, with no changes in abortion restrictions or protections since *Dobbs*. The implications of *Dobbs* and the 2024 election results on patient experience of PUL and diagnostic uncertainty has been not been well studied. One recent study explored clinician-reported implications of *Dobbs* on PUL and ectopic pregnancy management (20). We are currently conducting qualitative research with clinicians to understand the implications of *Dobbs* for PUL management. Future work is needed to understand how this impacts early pregnancy experiences for pregnant people.

### Implications for Practice

At our institution, the availability of a direct resident-staffed phone available day and night tended to improve participants’ overall impression of their care and provided access to direct medical communication on-demand. This dedicated phone line may be an actionable item at other academic centers with the resources to implement a similar system. While many participants reported positive experiences with the Beta Book phone, care provided by residents during this period of PUL monitoring may also be enhanced with focused training around the potential negative impact of diagnostic uncertainty on patients and approaches to improve communication in this period.

The need for access to more timely care or a sense of urgency from the care team arose as another primary actionable finding. For many participants, there was a dissonance between the serious threat of ectopic pregnancy as described to them and the slow pace of the care they received, particularly in the ED. Further, there were multiple complaints about the physical environment of the ED itself. Care efficiency for PUL could be significantly improved in settings outside of the ED, such as an EPAC setting. Several participants appeared unaware of EPAC availability prior to presenting to the ED, indicating a need for improved visibility of this service and call center optimization to appropriately direct patients to EPAC services. EPACs have been shown to reduce cost, wait time, and number of visits required for early pregnancy management (21–25).

While EPACs are intended to provide a space for expedited access to early pregnancy care as a safe and efficient alternative to the ED (3), one prior study showed that ED utilization did not decrease with EPAC availability, perhaps indicating that patients may sometimes seek more immediate care when having first trimester complications, and that ED services should also be optimized to meet early pregnancy needs (4). Furthermore, some participants voiced a desire for early pregnancy emergency care to occur in a dedicated pregnancy unit (i.e. incorporating early pregnancy emergency care into the labor & delivery triage setting). At our institution, patients are evaluated in the ED prior to 16 weeks gestation and on labor & delivery triage after that time. Our findings suggest that some patients may prefer for all emergency pregnancy care to occur in a dedicated hospital-based unit, separate from the ED.

### Strengths and Limitations

We used an inductive qualitative methodology in our exploration of themes around healthcare interactions regarding PUL, a common but understudied clinical experience. This study had limitations, including inclusion of only English-speaking patients, likely underrepresenting the large Hispanic and Asian immigrant communities in our region. Future research should focus on the PUL experiences and needs of patients who speak languages other than English. Furthermore, our hospital is an academic institution equipped with a 24/7 resident-staffed telephone available to patients. However, these capabilities and outcomes may not be applicable to non-academic centers or institutions without sufficient resources, or the resident workforce required to maintain a continuous service line for patient contact. Discussions of race and identity may have been constrained by the positionality of the two white cisgendered women interviewers, whose biases and personal interests could have shaped the interpretation of participant responses during the inductive coding and theme development process, though the use of collaborative validation helped mitigate these effects. While we included questions around discrimination in our structured interview guide, most participants did not feel this played a role, and none of the participants indicated they felt that race affected their PUL care experience, though several recounted prior experiences with racism in healthcare. While experiences around identity did not emerge as a theme in this study, given the disproportionate burden of maternal morbidity and mortality on people of color (5,26,27) and the central role of racism in this phenomenon, the role of racial bias in early pregnancy care experiences merits further exploration.

## Conclusion

Our findings revealed the successes and gaps in current communication around PUL diagnosis and management, the need for timely care that prioritizes patient needs and acknowledges the urgency of their presentation, and a desire for optimization of care environment, perhaps in a dedicated pregnancy-related emergency care unit that exists as a complement to the EPAC. Improved communication and training around diagnostic uncertainty in early pregnancy may improve healthcare interactions. We also found that participants felt they needed sooner engagement with their primary OB/GYN in pregnancy. These findings can help inform the development of new pathways for improving care early in pregnancy.

## Supplementary Material

Supplementary Files

This is a list of supplementary files associated with this preprint. Click to download.

- PULSEsupplementalmaterialsBMC.docx

## Figures and Tables

**Table 1.** Participant demographics, pregnancy characteristics, and health service measures –November 2023 - March 2024 (N=20)

Demographic Characteristics	n (%)
**Age** *mean (SD)*	30.6 (6.02)
**Age**	
18–24	4 (20)
25–29	5 (25)
30–34	4 (20)
35–39	6 (30)
40–44	1 (5)
**Race/Ethnicity**	
Asian	0 (0)
Hispanic	1 (5)
Native American/Alaska Native	0 (0)
Non-Hispanic Black	12 (60)
Non-Hispanic White	7 (35)
**Marital status**	
Married	9 (45)
Single	11 (55)
**Insurance**	
Public	13 (65)
Private	7 (35)
Uninsured	0
**Zip code median annual household income ($)** - *median, IQR*	42,703 (38,741–69, 22)
**Zip code % poverty** - *median, IQR*	27.2 (15.4–32.4)
**Pregnancy History**	
Prior pregnancy	16 (80)
First pregnancy	4 (20)
**Pregnancy Desiredness**	
Desired	14 (70)
Not desired	1 (5)
Unsure	1 (5)
Not documented	4 (20)
**Pregnancy Planned**	
Yes	11 (55)
No	4 (20)
Not documented	5 (25)
**PUL Pregnancy Outcome**	
Miscarriage/Early pregnancy loss	7 (35)
Ectopic	1 (5)
Spontaneous resolution PUL	9 (45)
Live birth	2 (10)
Ongoing viable pregnancy at time of interview	1 (5)
**Presenting Location**	
Emergency Dept	16 (80)
Office/EPAC	4 (20)
**Travel distance (miles)** *mean (SD)*	5.9 (5.3)
**Drive time (minutes)** *mean (SD)*	19.7 (9.4)
**Public transit time (minutes)** *mean (SD)*	40.4 (20.5)
**Presenting Symptom**	
Bleeding	14 (70)
Abdominal/pelvic pain	3 (15)
Positive pregnancy test	2 (10)
Other	1 (5)
**Number of ED Visits**	
0	4 (20)
1	13 (65)
2	3 (15)
**Number of Outpatient Visits**	
0	5 (25)
1	7 (35)
2	6 (30)
3	2 (10)
**Number of Ultrasounds**	
0	0 (0)
1	12 (60)
2	8 (40)
**Number of days on beta book** *mean (SD)*	23.3 (11.8)

**Table 2.** Quantitative findings: key themes and representative quotes

Theme	Description	Quotes
Provider Communication		
**Diagnostic uncertainty**	Provider communications regarding diagnostic uncertainty, lack of transparency, and lack of final diagnosis closure	“I think there was definitely some times where I was confused. I was told about the pregnancy of unknown location. Um, and that didn’t really make sense to me in terms of like what that even meant. Um, I think they tried their best to explain to me what that was. But afterwards, like, my husband and I were googling what it actually meant to try and figure out like what we were dealing with.” (209)
		“It was a lot of uncertainty from [the providers] as to what my case was, and how to handle it, which was then passed along to me…they didn’t know how to deal with me, so I didn’t know how to react to any of it.” (209)
		“…I’m just sitting there dumbfounded, confused, clueless, and then still hoping, despite my tears, that I’m still potentially pregnant when I wish someone would have been preparing me for the fact that more than likely I’m not. Of course, there’s a still chance maybe some miracle.” (136)
		“You know, I actually left the hospital believing there was a chance I was still pregnant, you know, that they didn’t want to say that I no longer was.” (136)
		“I had to come to the conclusion on my own that I had, you know, lost the baby. No one actually said you just had a miscarriage.” (136)
		“It wasn’t.as comforting as it could have been. Um, I feel like the doctors more so talked outside of the room, versus more to me.” (189)
		“…they knew, like as soon as I came, but they didn’t want to, like, immediately say it. So they kind of like teeter around it.” (189)
		“And for this to be my third [PUL], no one has an answer for me why.” (189)
**Feelings around exploring pregnancy attitudes**	Provider communications regarding pregnancy attitude and planning	“It just felt like they were like moving towards termination like without me even saying that’s what I wanted.” (081)
		“It’s not like they told me to stop trying [to conceive] because that would have broke my heart.” (025)
		“When the doctor came in and he was like, ‘Oh, congratulations.’ I’m just like, ‘congratulations on what? Like I’m negative for COVID, is that what you’re saying?’…And I thought he was saying, ‘Congratulations. You’d don’t have COVID.’ That’s what I was hoping he was going to say. But when he pulled out that little stick and it was two lines, I’m just like.’Oh.’ I didn’t know what to say.” (147)
**Feelings of dismissal or support**	Patients feeling dismissed or supported by providers	“[I wanted] the doctors to show that they care more and be more helpful and not just throw the resources at us, like here’s the paper.” (030)
		“It kind of, it kind of, it kind of felt like nobody was worried until they looked really bad. And everybody tried to scramble and tell me to go to their emergency room.” (015)
		“And my pain level was extremely high. They didn’t give me no meds, no nothing. So I think that was one of the things that like, I was like, okay, so just [laughter], so like me telling you my pain level was, like, at a thousand. They’re like, ‘Okay. We’re going to discharge you.’“ (007)
		“I didn’t feel rushed at all and they made me feel comfortable. And once I calmed down they proceeded with all the medical stuff, which was really nice.” (068)
**Ultrasound Experience**	Experience presenting for ultrasound and in person interactions with ultrasound staff	“I kind of picked up when I have a good ultrasound, the tech normally talks to you. And when it’s a bad ultrasound, the tech doesn’t say anything. So before I was even confirmed by the doctor, I was already like, I already knew because the tech didn’t say anything the whole time.” (189)
**Beta Book interaction experience**	Text and phones call reminders and results	“I had very low expectations. I was like, “There’s no way that people are following this,” but they actually were and followed up on it and were very good about it. For me.” (068)
		“I think that was it was helpful again just having someone to kind of explain the process and follow up. And, um, it definitely, uh, felt, you know, um, that my care was being followed adequately. “ (065)
**Timely Access to Necessary Services**		
**Urgency/emergency**	Pertaining to the urgent nature or danger of participant’s PUL.	“And if something is that risky and that fatal to somebody, and that could be something that is like a cause of death to somebody, or like cause infertility, I feel like those should be like prioritized more.” (015)
		“My pain level was extremely high. They didn’t give me no meds, no nothing…so, me telling you my pain level was, like, at a thousand. They’re like, “Okay. We’re going to discharge you.” (007)
		“So I didn’t have a problem, but I do think, like, um, when I did have like the possibility of it being ectopic, my appointments were pushed out so far. It didn’t really make sense to me that if they thought that that’s what it was, that, like, it wasn’t like an immediate emergent thing that I had to get done. They just kept sending me to get bloodwork. Like, I was getting bloodwork, I think, every two days.” (015)
		“When they called, it was like a, a sense of urgency, like you need to go now. But which I feel like that’s what I mean, they you know, should have told me that, but I feel like there was no precautions taken because y’all already had a fear that this was what was happening, which y’all kind of waited till the last minute to see that my levels had dropped so severely. And y’all was like, oh, like you really like, it’s just, it was traumatic. That was like awful. I’m grateful that I’m like okay, though. But in the moment, I was scared like I thought I was going to die. “ (015)
**Timeliness**	Discussions about wait times for appointments and in the ED	“Maybe let it - like maybe it could be more frequent like updates like, you know, “We have a lot of people. We didn’t forget about you. We’re still here,” like instead of waiting hours and hours to hear like an update. I don’t know. Maybe just like acknowledging that they do understand I’m waiting. They do understand, you know, I don’t know. Just being acknowledged more often, I guess, could help or else you’re just stuck there… Or else you’re just stuck there just like twiddling your thumbs for hours and you’re like, “I don’t even know when the next update is,” rather than, you know, I don’t know. Just - just like an acknowledgment, I guess.” (081)
		“There was so much going on in the emergency room. A lot of people was coming in sick and they was like so busy. I was in there for at least like four or five hours just for them to tell me I was pregnant. Which usually if I go somewhere else it won’t really take that long, but it was packed and busy.” (147)
		“So I think that is always really frustrating, especially in early pregnancy is like you’re sad initially. There is a lot of uncertainty, a lot of just kind of like you want to confirm the pregnancy and make sure everything is okay. And I just feel like that has been really the weakest part of the whole system for me, is just having to then get my doctor involved to get scheduling, to actually schedule the appointment.” (065)
**Counseling and grief support**	Provider’s ability or inability to provide counseling and grief support	“It’s an emergency room, so you feel like they don’t have time to tackle anybody’s feelings.” (112)
		“They give you pamphlets in regards to things like if something bad happens that they’re not held liable and - but yet no pamphlets on how to deal with your actual experience.” (136)
**Care Environment**		
**Care setting/Physical space**	Physical space/conditions, attributes of the physical space that they were seen in that impacted their experience.	“But I think there were over a hundred people in the emergency room that night. There was like fecal matter and urine on the chairs. People were like lined up in stretchers and in wheelchairs. It was like cold out. It was just like people throwing up. [Laughs] It was really disgusting. There is really no other way to put it. My husband and I waited there for like an hour. And oh, there was a guy like in handcuffs. Like it was just crazy.” (065)
**Presenting location - reasons**	How participants selected their initial care-seeking location.	“I literally got home from the ultrasound and Googled emergency rooms near me. And that was the first one that popped up on my Google Maps, so that’s where we went” (209)
		“I first went to urgent care on [Street 1], um, because my insurance, if I go straight to the emergency room I have to pay a co-pay of $250.00. So I went to the, uh, urgent care to, you know, just try to get them to see what was going on. And they told me that if anything was really wrong they would send me over to the emergency room anyway, so I could pay the $40.00 to be there for 20 minutes, then being forced to go to the hospital anyway. So I just chose to leave and go to the hospital down the street.” (094)
		“that’s [hospital] the closest one to where I live.” (189)

## Data Availability

The deidentified qualitative date generated and analyzed during the current study are available from the corresponding author on reasonable request.

## Abbreviations

hCG: human chorionic gonadotropin
PUL: pregnancy of unknown location
EPAC: early pregnancy assessment clinic
